# Whispers in the Neck: Unmasking Cystic Metastases From Hidden Papillary Thyroid Carcinoma

**DOI:** 10.7759/cureus.57388

**Published:** 2024-04-01

**Authors:** Kelly Lee Pak Hup, Yan Yu Chai, Yi Jing Kaw, Ranveer Singh Gill, Phei Fern Wang

**Affiliations:** 1 Otorhinolaryngology, Hospital Tengku Ampuan Rahimah, Klang, MYS; 2 Pathology, Hospital Tengku Ampuan Rahimah, Klang, MYS

**Keywords:** thyroidectomy, metastases, lymph node, cystic neck mass, papillary thyroid carcinoma

## Abstract

Papillary thyroid carcinoma is the most common thyroid malignancy and it frequently causes lymph node metastases. Approximately 50% of patients with papillary thyroid carcinoma have cervical lymph node metastases at the time of their initial presentation. Here we report a case of a young female who presented with a benign-appearing cystic neck mass, which was revealed to be metastases from occult papillary thyroid carcinoma. Completion thyroidectomy and neck dissection were done after the diagnosis of papillary thyroid carcinoma.

## Introduction

Papillary thyroid carcinoma is the most common thyroid malignancy attributed to about 80% of all thyroid cancers, occurring mainly in the third and fourth decades of life [1,2]. A solitary thyroid nodule is the most common clinical presentation. Purely cystic nodules as the presentation of papillary thyroid carcinoma are rare (<2% of all nodules) [3]. Cystic swellings in the anterior or posterior triangles of the neck are usually benign, especially in young adults. Nevertheless, they may be malignant occasionally; therefore, among the differential diagnoses, cystic cervical metastasis from an occult thyroid papillary carcinoma should be considered [3-5]. We report a case of a young female who initially presented with a painless cystic neck mass without a clinically apparent thyroid nodule. The excision of the cystic neck mass and a diagnostic hemithyroidectomy revealed metastatic papillary thyroid carcinoma. The aim of this case report is to emphasize that papillary thyroid carcinoma should be considered as a differential diagnosis of a cystic neck mass and discuss the appropriate investigations and treatment for such cases.

## Case presentation

A 22-year-old lady with underlying young hypertension presented with a painless right-sided neck swelling that had increased in size over one year. Clinical examination revealed a right-sided neck swelling over the posterior triangle, measuring approximately 5 × 7 cm, firm in consistency, and nontender. Flexible nasopharyngolaryngoscopy was otherwise normal. Ultrasound examination of the neck revealed an ill-defined, wider-than-tall, solid, hypoechoic lesion with punctate calcification seen within the lower pole of the right thyroid gland measuring 1.5 cm × 1.3 cm × 2.3 cm (AP × W × CC). There was also an anechoic lesion seen at the right neck close to the angle of the right mandible measuring 2.3 cm × 4.7 cm × 2.5 cm. The overall impression was a TIRADS 5 right thyroid lesion and a right neck cystic lesion of indeterminate origin.

**Figure 1:**
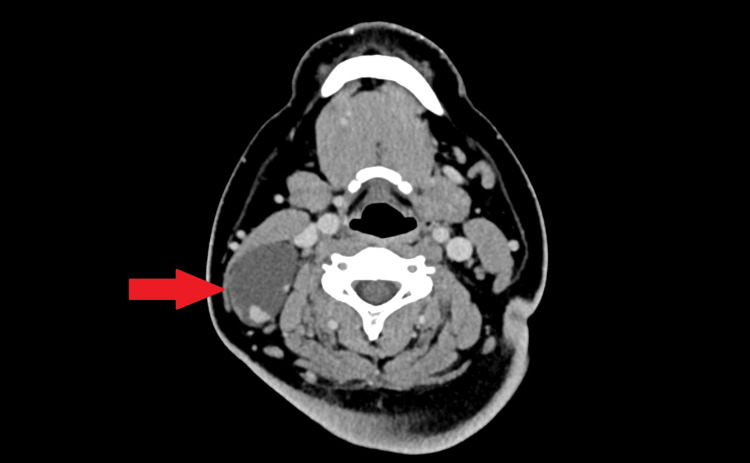
Note the thin internal septation with an enhancing soft-tissue component within.

**Figure 2:**
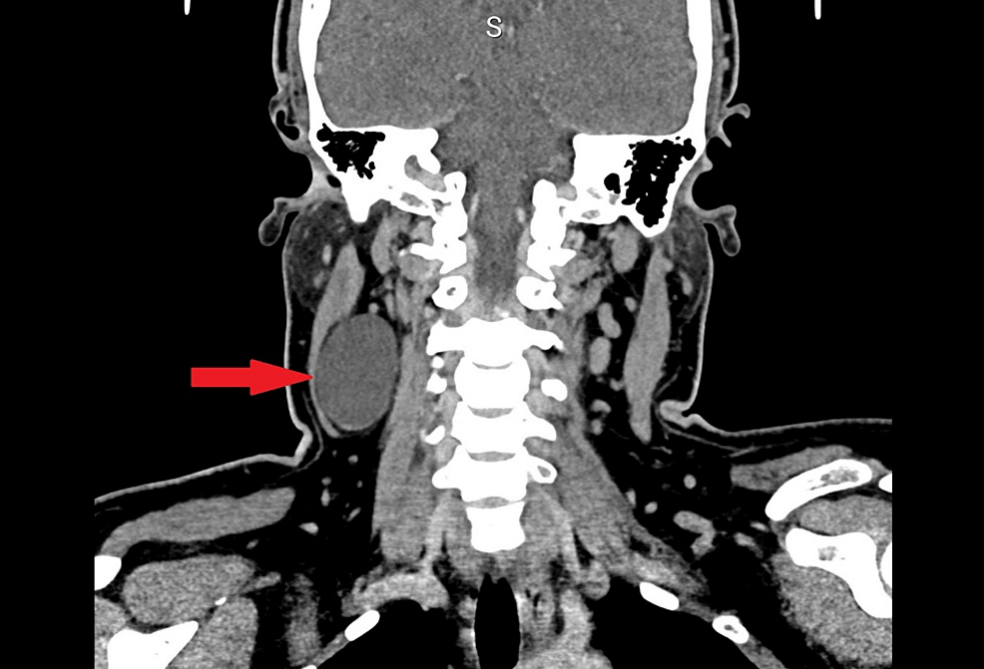
A well-defined, lobulated, thin-walled hypodense lesion posterior to the right sternocleidomastoid muscle at the mid-neck region.

**Figure 3:**
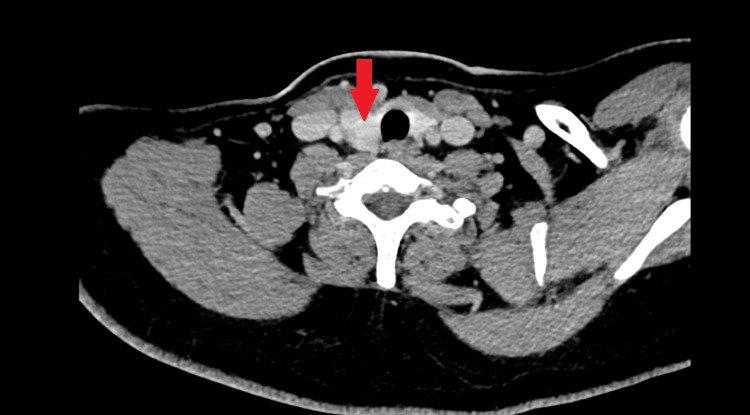
Right thyroid nodule was seen.

CT scan reported a well-defined, lobulated, thin-walled hypodense lesion posterior to the right sternocleidomastoid muscle at the mid-neck region which was reported as a branchial cleft cyst, and a right thyroid nodule was also seen as well; however, upon closely reviewing the CT images, an enhancing component was noted within the cyst. This CT finding is shown in Figures 1-3.

This gave rise to a suspicion that the cystic mass may represent a cystic metastasis from an occult papillary thyroid carcinoma. Ultrasound-guided fine-needle aspiration cytology (FNAC) of both cyst and thyroid lesion was unsatisfactory for evaluation. In view of this, the patient was then planned for a right hemithyroidectomy and excision of the right neck cyst to obtain a definitive diagnosis.

**Figure 4:**
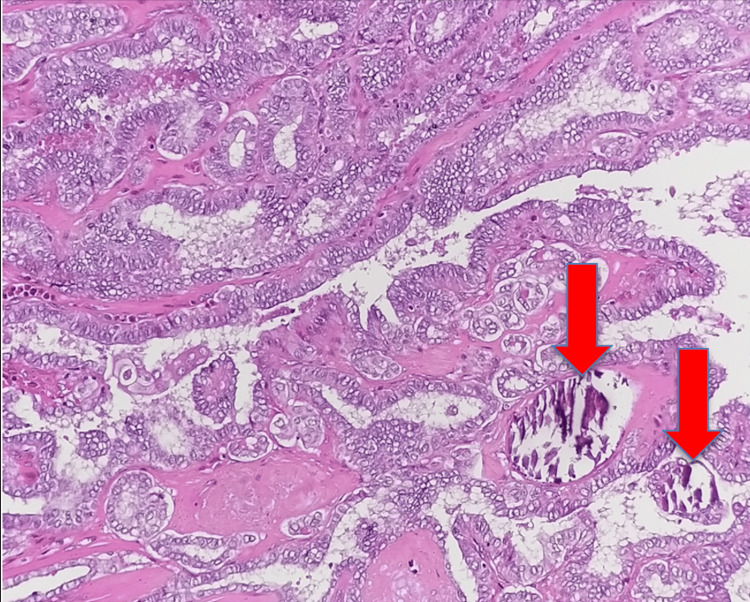
Papillary architecture with classical PTC nuclear features. Psammoma bodies can be seen (shown by red arrows) (H&E, x10). PTC, papillary thyroid carcinoma.

**Figure 5:**
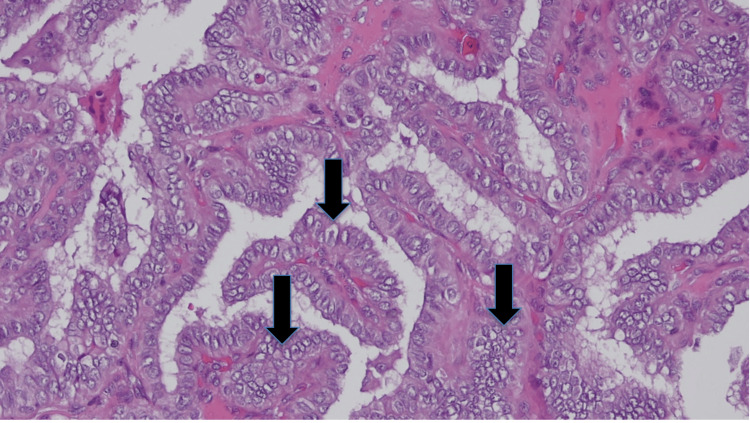
Thyroid tumor exhibiting papillary architecture with fibrovascular cores lined by epithelial cells featuring enlarged, elongated, and overlapping nuclei with optically clear chromatin also known as "Orphan Annie nuclei" (shown by black arrows) (H&E, ×20).

**Figure 6:**
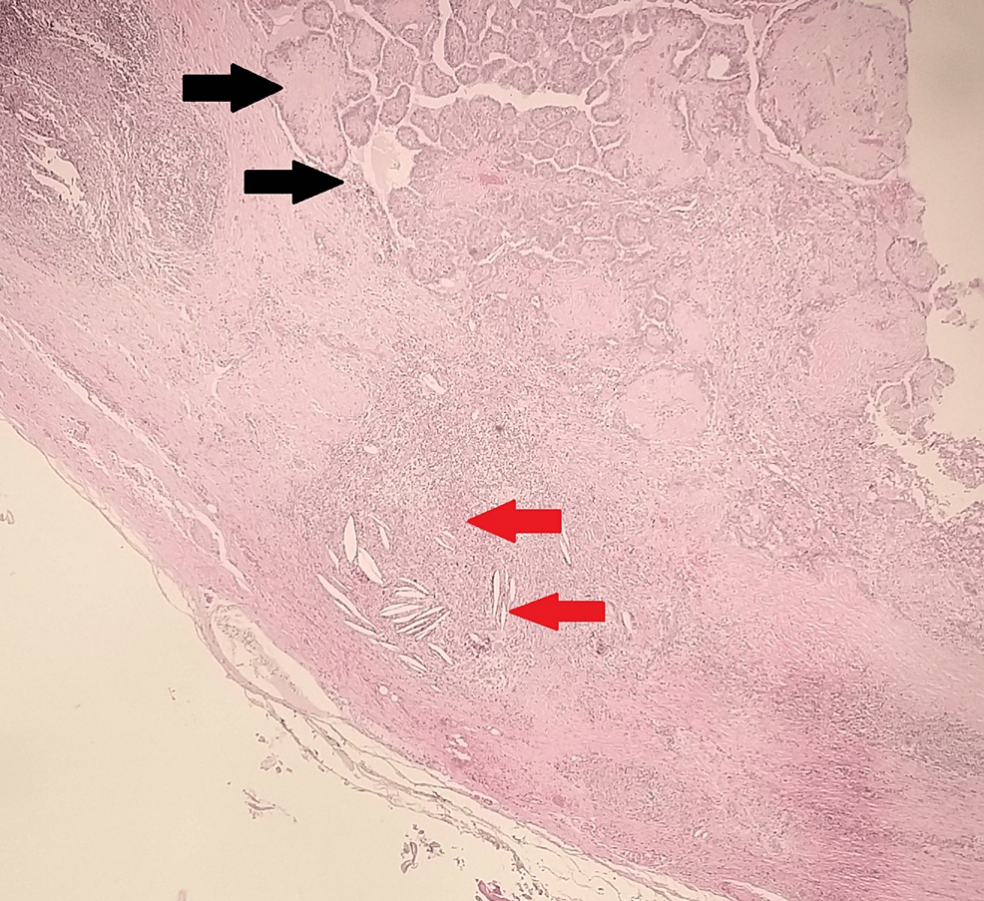
The cystic mass shows tumor cells with papillary configuration (shown by black arrows) and adjacent foreign-body-type reaction featuring cholesterol clefts, clusters of foreign-body-type multinucleated giant cells and lymphoplasmacytic cells (shown by red arrows) (H&E, ×10).

**Figure 7:**
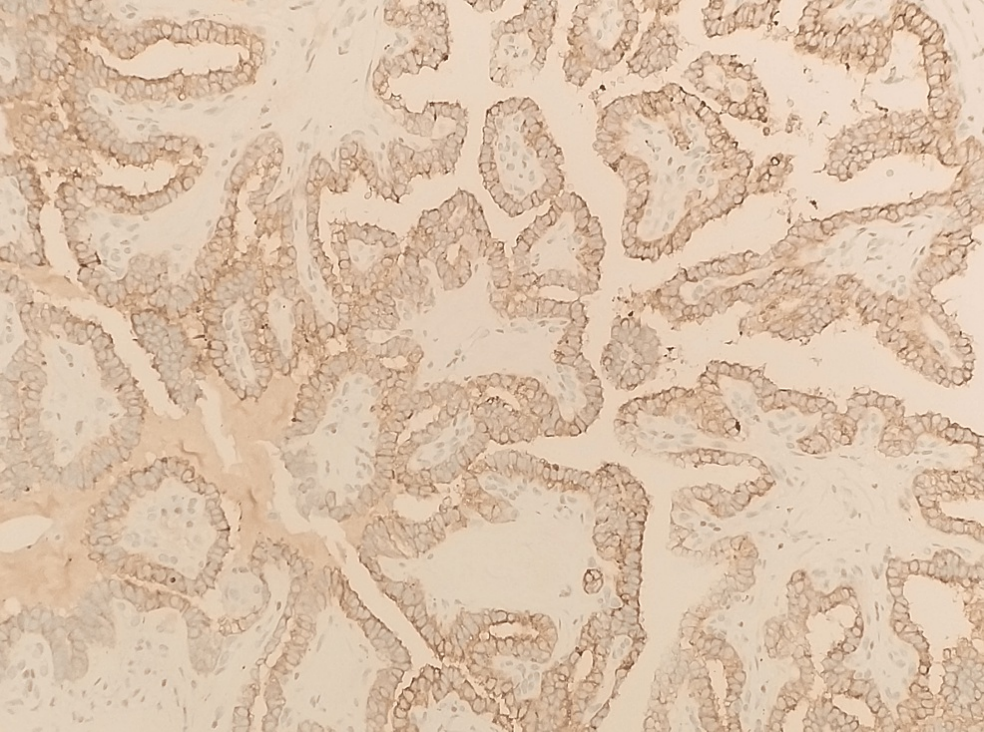
The tumor cells within the cystic mass express thyroglobulin (IHC, ×4). IHC, immunohistochemistry.

The histopathology reported the thyroid lesion as papillary thyroid carcinoma, and the neck cyst as having features favoring cystic metastatic papillary thyroid carcinoma. Microscopic evaluation revealed psammoma body-like calcifications, Orphan Annie nuclei, cholesterol clefts, and expression of thyroglobulin. These microscopic features are shown in Figures 4-7.

This was consistent with metastatic papillary thyroid carcinoma.

A PET scan did not show any F-fluorodeoxyglucose (FDG)-avid tissue suggestive of local recurrence or nodal or distant metastasis. A completion thyroidectomy, right modified radical neck dissection, and left selective neck dissection were subsequently carried out. Histopathological examination of the neck dissection specimen was consistent with papillary thyroid carcinoma with positive right level II and III neck nodes. The overall staging was T1bN1bM0 (stage II).

## Discussion

The most common benign neck cysts are branchial cleft cysts, dermoid cysts, teratoma, epidermoid cysts, and cystic hygromas [6]. As evidenced in our case report, the patient presented with a benign-appearing lateral neck cyst, with the absence of any constitutional symptoms. A high index of suspicion and closely scrutinizing the CT images gave the impression of cystic lymph node metastases from an impalpable occult thyroid papillary carcinoma. Therefore, it is paramount to rule out the presence of thyroid cancer in patients presenting with an otherwise asymptomatic neck cyst, as it may subsequently lead to a reduction in mortality rate [5,7]. The prevalence of occult thyroid malignancy in patients with lateral neck cysts is approximately 11%, with a mean age of 29 years [8]. Approximately 40% of all lymph node metastases from papillary thyroid carcinomas have the tendency to mimic the features of a benign neck cyst. This may result from cystic degeneration of a thyroid primary or true malignant transformation of ectopic thyroid tissue [9].

For patients presenting with a neck mass, triple assessment should be employed including clinical examination of the head and neck, an FNAC of the neck mass, and imaging with ultrasound or CT scan.

Ultrasound is a very useful tool for the detection and assessment of thyroid gland lesions and cervical lymphadenopathy [9,10]. Sonographic features that are suggestive of malignancy include a predominantly solid composition, hypoechogenicity, absence of a hypoechoic halo, presence of microcalcifications, irregular margins, and increased vascularity [11]. In small thyroid lesions, ultrasound may have a limited role [12]. CT is the best modality for staging lymph node involvement and for overall staging of papillary thyroid carcinoma [13].

Fine-needle aspiration may help in the differential diagnosis of neck masses. However, FNAC is less sensitive in the diagnosis of cystic neck masses compared to solid masses with a false-negative rate ranging from 50% to 67% [5]. According to the World Health Organization's Classification of Tumours, the histology of papillary thyroid carcinoma consists of nuclear features showing an enlarged, oval-shaped, elongated and overlapping appearance [14]. Fine-needle aspirates in the case of cystic metastases of papillary thyroid carcinoma are often red or brown in color because of thyroglobulin. To aid in confirming the diagnosis, the aspirate can be sent for thyroglobulin assay [15].

Once a diagnosis of thyroid cancer has been established, adequate surgery by excision of the cyst, total thyroidectomy, and neck dissection is crucial for a good prognosis. Postoperative radioiodine ablation of thyroid remnant is recommended to reduce recurrences [4].

In conclusion, a strong clinical suspicion is essential in the investigation of solitary neck masses despite an apparently benign course such as a branchial cyst. Malignant conditions such as occult papillary thyroid carcinoma must be taken into consideration [10].

## Conclusions

Most papillary thyroid carcinomas present with solitary thyroid nodules. However, they may have come with an unusual presentation; for example, a solitary cystic nodal mass or multiple cystic masses as presented in this case report. Hence, strong clinical suspicion is essential in the investigation of solitary neck masses despite an apparently benign course such as a branchial cyst. Malignant conditions such as occult papillary thyroid carcinoma must be taken into consideration. Once the diagnosis of papillary thyroid carcinoma has been established, adequate surgery by completion thyroidectomy and neck dissection is crucial for a better prognosis.
